# Primary *Toxoplasma gondii* infection-associated with hemophagocytic syndrome in a man with HIV infection: a case report

**DOI:** 10.1186/s12879-021-07022-6

**Published:** 2022-01-06

**Authors:** Ying Zhou, Yongfeng Liu, Ying Wen

**Affiliations:** 1 Department of Infectious Diseases, The First Affiliated Hospital of China Medical University, No. 155, Nanjing North Street, Heping District, Shenyang, 110001 Liaoning Province China; 2 BGI Pathogenesis Pharmaceutical TechnologyBGI-Shenzhen, Shenzhen, Guangdong Province China

**Keywords:** Primary *Toxoplasma gondii* infection, Hemophagocytic syndrome, Human immunodeficiency virus, Metagenomic next-generation sequencing

## Abstract

**Background:**

Reactivation of latent *Toxoplasma gondii* (*T. gondii*) infection is more common than primary infection in patients with human immunodeficiency virus (HIV). We report a rare case of primary *T. gondii* infection-associated hemophagocytic syndrome (HPS).

**Case presentation:**

A man with HIV infection presented with fever, dyspnea and pancytopenia. He was diagnosed with primary *T. gondii* infection by the seroconversion from single-positive IgM antibody to double-positive IgM and IgG antibody. Metagenomic next-generation sequencing (mNGS) of a plasma sample yielded high reads of *T. gondii* DNA. He responded well to combined anti-*Toxoplasma* medicines and glucocorticoid treatment.

**Conclusions:**

In patients with HPS and positive *T. gondii* IgM antibody, mNGS analysis of a peripheral blood sample is helpful in diagnosing disseminated *T. gondii* infection. The dynamic changes by serological detection for IgM and IgG of *T. gondii* further supported the inference that the patient has experienced a primary *T. gondii* infection.

## Background

In individuals with human immunodeficiency virus (HIV) infection, reactivation of latent *Toxoplasma gondii* (*T. gondii*) infection is more common than primary infection [1]. Primary *T. gondii* infection is usually asymptomatic. The central nervous system is mostly affected in a *T. gondii* reactivation infection. Cases of hemophagocytic syndrome (HPS) associated with disseminated toxoplasmosis infection have been reported among transplant recipients and immune competent adults [2, 3]. Among individuals with HIV infection, the major causes of HPS are infection with *Mycobacterium*, Cytomegalovirus, *Cryptococcus neoformans*, and hematological or tumoral disease [4]. However, cases of primary *T. gondii* infection-associated HPS have not been reported in HIV-infected individuals. Here, we described a man with HIV infection and HPS, who was ultimately diagnosed with primary *T. gondii* infection.

## Case presentation

A 33-year-old Chinese man with HIV infection was admitted to our hospital on October 30, 2019. The patient presented a 15-day history of sustained fever (the highest temperature was 39 ℃), followed by 2 days of dyspnea but without impaired consciousness, headache, rash and weight loss. He had been previously diagnosed with HIV infection and had been taking antiretroviral therapy (ART) with a regimen of lamivudine, tenofovir, and efavirenz for 6 years. He reported that his CD4 + T-cell count had increased from 100 to 200 cells/μL 2 years previously.

**Fig. 1:**
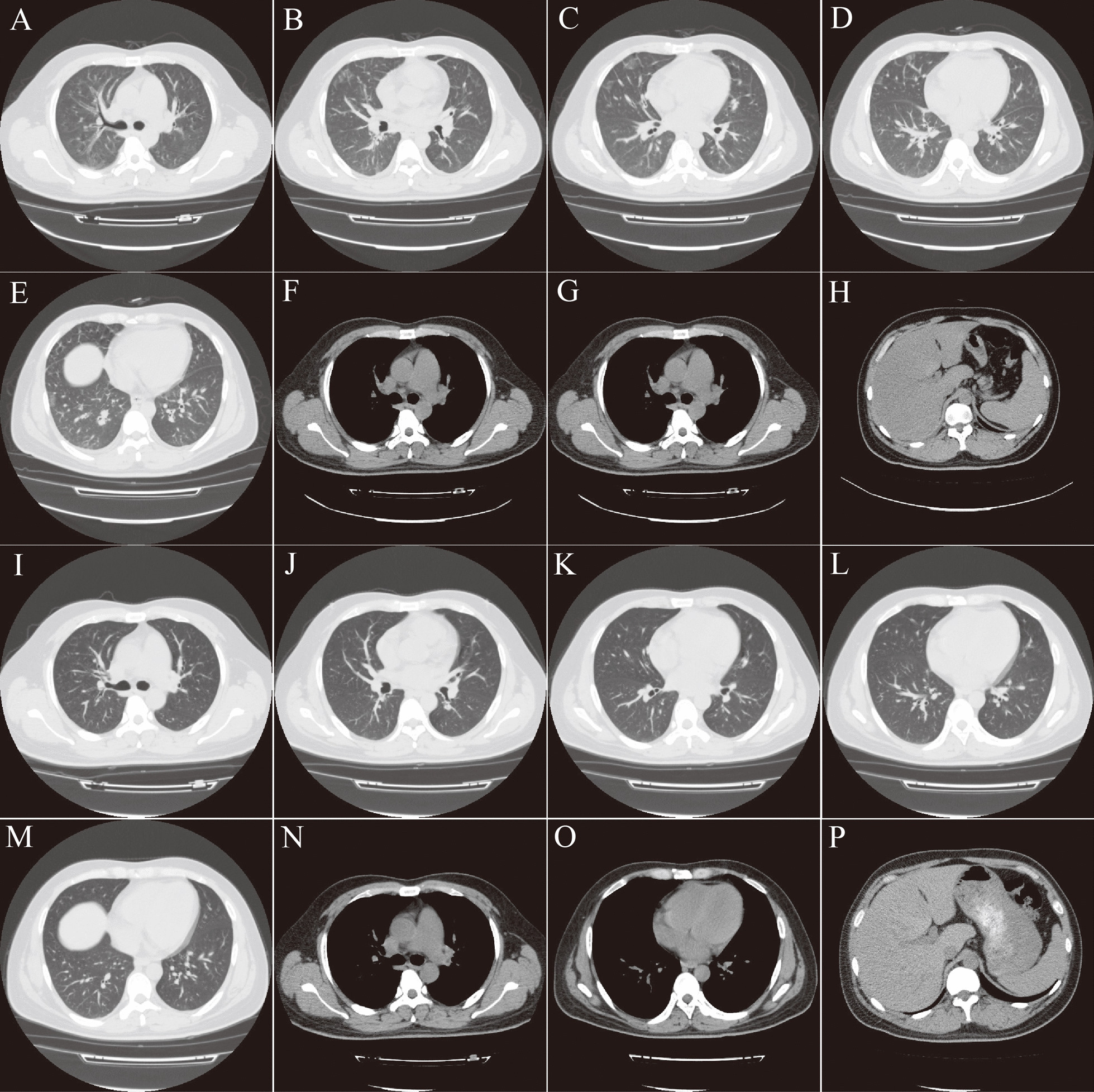
Presentation of Chest and Abdomen computed tomography (CT) findings. Chest CT showed thickened bronchial walls with surrounding interstitium in both lungs, double-track signs (**A**), thickened interlobular septa, multiple ground-glass opacities in the periphery of both lungs (**B**–**E**), widened pulmonary artery segment (**F**), a small amount of pleural effusion on both sides and pericardial effusion (**G**). Abdomen CT showed severe fatty liver and splenomegaly (**H**). After 2-week chest CT indicated significant improvement of pulmonary lesions (**I**–**M**), normal pulmonary artery segment is visible (**N**), pleural effusion and pericardial effusion disappeared (**O**), fatty liver and normal spleen (**P**)

Chest computed tomography (CT) showed thickened bronchial walls with surrounding interstitium in both lungs, double-track signs, thickened interlobular septa, multiple ground-glass opacities in the periphery of both lungs, widened pulmonary artery segment, a small amount of pleural effusion on both sides and pericardial effusion. Abdomen CT showed severe fatty liver and splenomegaly (Fig. 1A–H). No abnormalities were detected on contrast magnetic resonance imaging (MRI) of the brain.

Laboratory results revealed normal leukocyte count, decreased platelet count (52 × 10^9^/L), mild anemia (99 g/L), elevated level of C-reactive protein (104.5 mg/L), glutamic amino transferase (121 U/L), aspartate amino transferase (193 U/L), lactic dehydrogenase (LDH) (above 4300 U/L), and a normal galactomannan level. His CD4 + T-cell and CD8 + T-cell counts were only 8 cells/μL and 510 cells/μL, respectively. Arterial blood gas analysis revealed hypoxemia (arterial oxygen tension 55.2 mmHg). He tested negative for the serum cryptococcal antigen test, and IgM antibodies to herpes simplex virus, Epstein-Barr virus (EBV), and cytomegalovirus (CMV), but was positive for IgG antibodies. The EBV and CMV DNA in the peripheral blood were undetectable. Three sets of blood cultures for bacteria and fungi were negative.

**Fig. 2:**
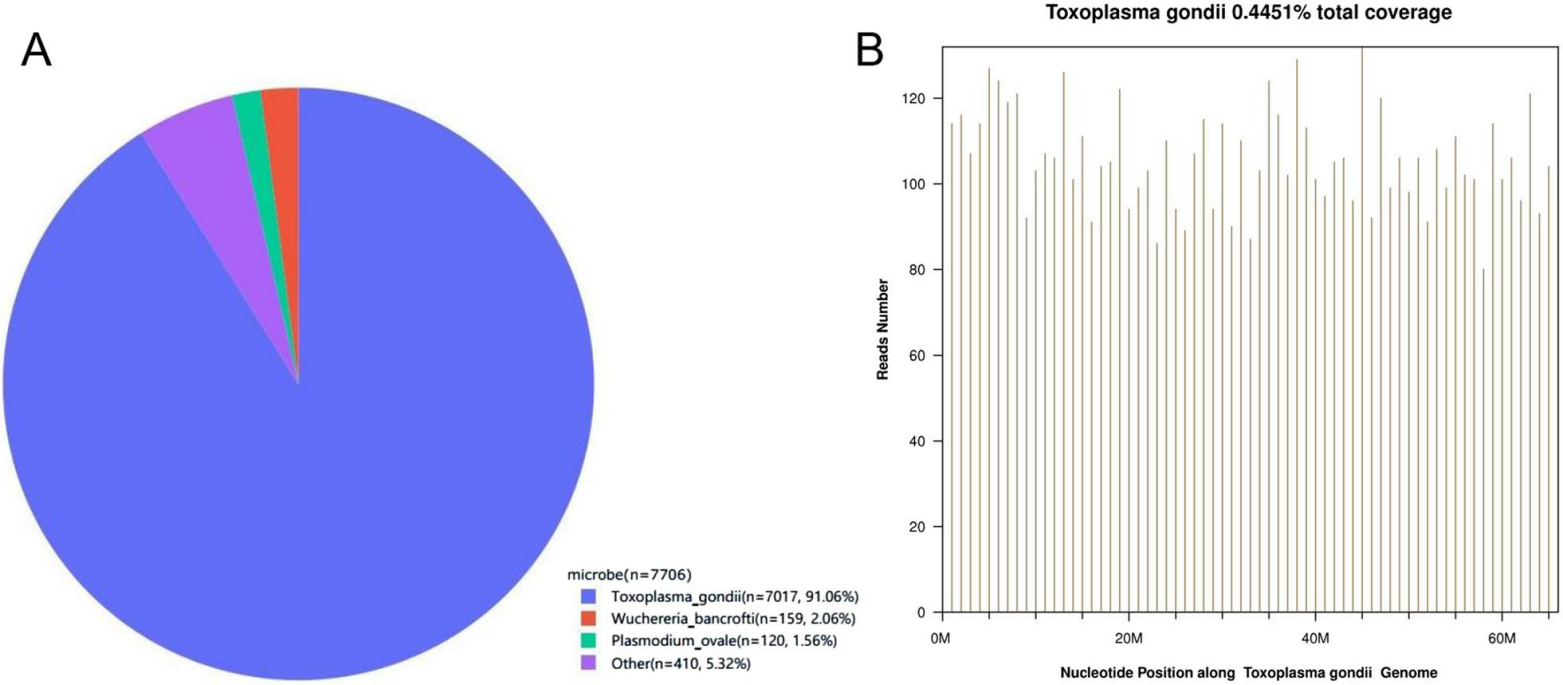
Pathogen identification from blood samples using next-generation sequencing method. The number of sequencing mapped reads that corresponded to *T. gondii* was 7017 (91.06%) (**A**) with 0.44514% genome coverage (**B**). Reads distribution of total DNA sequence in the sample was without the human host

The patient was positive for *T. gondii* IgM antibody (9.82 AU/mL; reference range: 0–6 AU/mL) (TOXM0460DS/96Wells, ELISA, DiaSorin) and negative for IgG antibody (7.04 IU/mL; reference range: 0–7.2 IU/mL) (TOXG0460DS/96Wells, ELISA, DiaSorin). The HIV RNA load was 3.1 × 10^5^ copies/mL and drug resistances were detected. His acute respiratory failure was assumed to be caused by *Pneumocystis jirovecii* (*P. jirovecii*) pneumonia (PJP). After 5 days treatment with sulfamethoxazole (SMZ; 15 mg/kg/day), trimethoprim (TMP; 15 mg/kg/day), and methylprednisolone (80 mg/day), his fever and dyspnea showed no improvement. Subsequently, a diagnosis of HPS was considered based on the following criteria: sustained fever; pancytopenia (leukocyte count: 2.51 × 10^9^/L, platelet count: 9 × 10^9^/L, hemoglobin: 8.5 g/dL); elevated levels of sCD25 (1485 U/mL; reference range: 223–710 U/mL), serum ferritin (> 2000 μg/L), and triglyceride (4.74 mmol/L); splenomegaly; and a decreased CD16 + CD56 + T-cell count (58 cells/μL; reference range: 90–590 cells/μL). Two attempts of bone marrow biopsy failed to obtain any bone marrow components. A plasma sample was sent to BGI PathoGenesis Pharmaceutical Technology (BGI-Shenzhen) for metagenomic next-generation sequencing (mNGS), which indicated *T. gondii* infection (Fig. 2).The patient was treated with a 5-day course of human immunoglobulin (0.4 g/kg/day) and combined therapy of SMZ-TMP, clindamycin and methylprednisolone. The ART drugs were changed to a regimen of lopinavir/ritonavir and dolutegravir sodium. A week later, fever and dyspnea were relieved, his platelet count recovered to 50 × 10^9^/L, serum *T. gondii* IgM antibody had increased to 12 AU/mL and IgG antibody had turned positive (7.49 AU/mL), which confirmed the diagnosis of primary *T. gondii* infection associated HPS. Two weeks later, his chest CT showed significant improvement (Fig. 1I–P). After 6 weeks of treatment, his red blood cell count had returned to normal. Four months later, *T. gondii* IgM antibody increased to over 160 AU/mL and IgG antibody increased to > 400 AU/mL. The CD4 + T-cell count was 169 cells/μL, CD8 + T-cell count was 2540 cells/μL, and HIV RNA load was 35 copies/mL.

## Discussion and conclusion

This was a rare case of primary *T. gondii* infection in an AIDS patient with HPS who previously experienced virological and immunological failure and had not been administered primary prophylaxis with SMZ-TMP. We were unable to find any published reports of primary *T. gondii* infection with HPS in patients with HIV. The primary *T. gondii* infection was initially identified based on the seroconversion from single-positive IgM antibody to double-positive IgM and IgG antibodies. However, the sensitivity, specificity and positive predictive values of IgM antibody by enzyme-linked immune sorbent assay for acute toxoplasmosis infection have been reported to be 98.1%, 65.0% and 43.3%, respectively [5]. False positive *T. gondii* IgM antibody can occur due to cross-reactivity with various autoimmune antibodies [6], while false negative *T. gondii* IgG antibody can occur in immune compromised individuals [7]. The diagnosis of disseminated *T. gondii* infection was subsequently confirmed by mNGS of a plasma sample. To our knowledge, only two cases HIV-infected patients with *T. gondii* infection-associated HPS have previously been reported, and both were due to reactivated *T. gondii* infection with positive IgG and negative IgM antibodies [8, 9]. Reactivation of latent *T. gondii* infection is more common than primary infection in patients with HIV, and most reported cases of primary *T. gondii* infection were from kidney transplant recipients [2]. In HIV-infected individuals, regardless of their immune status, primary *T. gondii* infection is generally asymptomatic [4, 10, 11], although some reported manifestations of macular rash [12] or acute respiratory distress [13]. In China the seroprevalence of *T. gondii* infection was 8.2% from 2000 to 2007, but has increased in recent years [14]. The prevalence of *T. gondii* IgG antibody in individuals with HIV infection has been reported to be higher than that of healthy controls (9.7% vs. 4.7%), while the seroprevalences of *Toxoplasma* IgM antibody is similar in both groups [15].In this patient, the level of *T. gondii* IgM and IgG antibodies increased greatly in the convalescent period. Generally, *T. gondii* antibody levels returned to normal within 4–6 months; however, some cases reported persistence of positive *T. gondii* IgM longer than 1 year. In this case, the delayed disappearance of *T. gondii* IgM antibody may be associated with obvious immune function restoration from HPS and virological control after adjusting the regimen of ART. Notably, serum *T. gondii* antibodies were only tested at baseline, 1 week, and at 4 months, possibly missing the peak level of *T. gondii* IgM antibody.

The lung is the second most common site of *T. gondii* infection. In transplant recipients and individuals with HIV infection, respiratory manifestations of acute toxoplasmosis usually present as a subacute febrile illness with cough and dyspnea. Patients with *T. gondii* pneumonia have significantly higher LDH levels than those with PJP. The diagnosis of pulmonary toxoplasmosis is challenging due to the nonspecific nature of clinical and radiographic findings. Chest CT may reveal centrilobular patchy or diffuse ground-glass opacities resembling PJP. Extrapulmonary involvement may be present in the liver, brain, bone marrow, heart, and stomach. Pulmonary coinfection of *T. gondii* and *P. jirovecii* has been reported [16]. Although no bronchoalveolar lavage fluid (BALF) specimen was tested in this case, the blood mNGS did not detect *P. jirovecii*, suggesting that the patient had *T. gondii* monoinfection without *P. jirovecii* coinfection.

Based on the brain MRI, neurological manifestations and lesions were not obsverved in the patient. In patients with toxoplasmic encephalitis (TE), MRI commonly shows one or more focal ring-enhancing mass lesions, but TE can manifest as diffuse encephalitis or ventriculitis. Moreover, there have been reports of unmasking and paradoxical TE immune reconstitution inflammatory syndrome after initiating ART in patients with HIV infection [17, 18].

Among different specimens, hematoxylin and eosin stainings, immunoperoxidase staining, and polymerase chain reaction (PCR) can be used for the detection of *T. gondii*. However, PCR detection for *T. gondii* was unavailable in our hospital. In this patient, cerebrospinal fluid (CSF), bone marrow specimen, and BALF specimen were not obtained. Patients with TE can be diagnosed by mNGS using CSF specimen [19]. However, to our knowledge, there have been no previous reports of mNGS being used to diagnose toxoplasmosis using a peripheral blood sample.

In summary, *T. gondii* infection-associated HPS can occur in primary and reactivated latent infection. The application of mNGS analysis using a peripheral blood sample could help in the diagnosis of *T. gondii* infection when it is difficult to obtain optimal specimens. Early diagnosis and treatment with the combination of anti-*Toxoplasma* and glucocorticoid medications can help in achieving favorable outcomes. In *T. gondii* seronegative patients with severe immunodeficiency, prevention of primary infection using SMZ-TMP prophylaxis is recommended.

## Data Availability

All data generated or analyzed during this study are included in this published article.

## Abbreviations

ART: Antiretroviral treatment
BALF: Bronchoalveolar lavage fluid
CMV: Cytomegalovirus
CrAg: Cryptococcal antigen
CSF: Cerebrospinal fluid
CT: Computed tomography
EBV: Epstein-Barr virus
HIV: Human immunodeficiency virus
HPS: Hemophagocytic syndrome
LDH: Lactic dehydrogenase
mNGS: Metagenomic next-generation sequencing
MRI: Magnetic resonance imaging
PJP: *Pneumocystis jirovecii* Pneumonia
SMZ: Sulfamethoxazole
TE: Toxoplasmic encephalitis
TMP: Trimethoprim
